# Beyond the Spine: Exploring Mental Health Disorders in Spondylodiscitis

**DOI:** 10.3390/jcm14061905

**Published:** 2025-03-12

**Authors:** Julius Gerstmeyer, Anna Gorbacheva, Clifford Pierre, Neel Patel, Donald David Davis, Tara Heffernan, Periklis Godolias, Tobias L. Schulte, Thomas A. Schildhauer, Amir Abdul-Jabbar, Rod J. Oskouian, Jens R. Chapman

**Affiliations:** 1 Swedish Neuroscience Institute, Swedish Medical Center, 550 17th Avenue, Suite 500, Seattle, WA 98122, USA; 2 Department of Orthopedics and Trauma Surgery, St. Josef Hospital Bochum, Gudrunstraße 56, 44791 Bochum, Germany; 3 Department of General and Trauma Surgery, BG University Hospital Bergmannsheil, Bürkle-de-la-Camp-Platz 1, 44789 Bochum, Germany; 4 Seattle Science Foundation, 550 17th Avenue, Suite 600, Seattle, WA 98122, USA; 5 Department of Orthopedics and Trauma Surgery, St. Josef Hospital Essen-Werden, Propsteistrasse 2, 45239 Essen, Germany

**Keywords:** infection, mental health disorder, spondylodiscitis, spine surgery, readmission rate

## Abstract

**Background/Objectives**: Spondylodiscitis (SD) is a challenging and multifaceted condition with increasing incidences globally. Mental health disorders (MHDs) are well recognized for their negative impacts on outcomes. To our knowledge, the effects of MHDs on SD have not been studied. This study aims to assess the incidence of MHDs in patients hospitalized for SD, and their impact on 90-day all-cause readmission rates using the Nationwide Readmission Database (NRD). **Methods**: A retrospective analysis using the 2020 NRD was performed. Adult patients were selected by primary ICD-10 codes for SD. MHDs were defined by ICD-10 F-codes. Demographic and clinical data were extracted, and readmissions were identified using VisitLinks. Patients were stratified based on MHD presence, with statistical analyses conducted to identify independent risk factors for readmission. **Results**: Of a total of 6139 patients, 3771 (61.4%) had an MHD. The overall 90-day readmission rate was 35%, with MHD patients experiencing a significantly higher rate (36.1%). Substance-related disorders, particularly opioid (OR 1.187, *p* = 0.019) and alcohol use disorders (OR 1.310, *p* = 0.020), were independently associated with increased readmission risk. Although common, depression, anxiety, schizophrenia, and personality disorders were not significant predictors. **Conclusions**: MHDs are prevalent among SD patients and are associated with an increased risk of hospital readmission, particularly in those with substance-related disorders. Integrating mental health interventions into SD management may improve patient outcomes. This study is limited by the use of an administrative database, which may lead to potential under-reporting of clinical variables. Future research may explore targeted interventions to optimize care for this high-risk population.

## 1. Introduction

Spondylodiscitis (SD) is a primary infection of the intervertebral disc with secondary infection of the vertebrae. Worldwide SD incidence has increased in recent decades, with Germany observing an increase of 41.6% during that time frame, causing increasing clinical concern [1,2,3,4]. This increase was previously attributed to improvements in diagnostic tools such as neuroimaging techniques and an aging and increasingly multimorbid population. Despite advancements in diagnostic and treatment modalities, SD still remains challenging to manage, with reported mortality and readmission rates of up 23% and 40.1%, respectively. This is particularly striking when compared to the significantly lower readmission rate of 2.3% following elective lumbar spine surgery [5]. The rates in SD are influenced by multiple factors, including the causative pathogen, treatment, and infection severity, such as the presence of epidural abscesses [6,7,8,9,10]. Risk factors for the development of SD include various medical comorbidities, as well as chronic alcohol and drug dependency [4,11]. 

Mental health disorders (MHDs), including substance related disorders, are an important covariable related to outcomes in spine surgery. Several studies have demonstrated that patients with MHDs, especially with depression and anxiety, have an increased likelihood of experiencing perioperative adverse events, worse functional outcomes, and dissatisfaction following surgery [12,13,14,15]. It is increasingly recognized that MHDs also adversely impact psychoneuroimmunological responses. Specifically, MHDs such as depression are associated with high levels of stress, increased systematic inflammation, and impaired immune responses due to high levels of cortisol. This may make patients more prone to infection [16,17,18]. In patients with spondylodiscitis, a high level of functional disability with high levels of depression and anxiety have been reported [19]. Moreover, the recent literature suggests that individual temperament profiles may influence patients’ ability to cope with illness and affect their adherence to medical treatment [20]. Behavioral factors commonly observed in MHDs patients, such as reduced healthcare-seeking behaviors, avoidance of medical appointments, and inconsistent medication use, may lead to prolonged infection durations and increased risks of complications. To our knowledge, the interdependence of MHDs and spondylodiscitis have not been studied. We hypothesize that MHDs increase the risk of 90-day hospital readmission in patients with SD. This study aims to assess the incidence of MHDs in patients hospitalized for SD, and their impact on 90-day all-cause readmission rates by utilizing the Nationwide Readmission Database. 

## 2. Materials and Methods

Using the 2020 Nationwide Readmissions Database (NRD), Healthcare Cost and Utilization Project (HCUP), Agency for Healthcare Research and Quality, a retrospective analysis was performed [21]. Adult patients (≥18 years) were selected for spondylodiscitis by primary International Classification of Diseases 10 (ICD-10) codes, Version 2023.1 (M46.2x, M46.3x and M46.4x). Demographic information and admission details (such as diagnosis, length of stay (LOS), in-hospital mortality, and readmission, as well as all comorbidities) were extracted. Mental health disorders were defined by the ICD-10 F-code system. It categorizes mental disorders into distinct groups, including mood disorders (F30–F39), anxiety and stress-related disorders (F40–F48), schizophrenia and psychotic disorders (F20–F29), substance-related disorders (F10–F19), and personality disorders (F60–F69). In this study, we included all mental health disorders within these categories. All causes of readmissions were identified by NRD *VisitLinks*. To ensure complete 90-day readmission assessment, we restricted our analysis to the first three quarters of 2020. Comorbidities were defined by ICD-10 codes and primary clinical classification software refined (CCSR) for ICD-10 category codes, Version 2023.1 [22]. To address the severity of medical comorbidities, the Elixhauser Comorbidity Index for in-hospital mortality and all-cause 30-day readmission were calculated for all patients [23]. As per the HCUP data use agreement, any comorbidities were excluded from tabulated comparison if numbers were 10 or lower.

Statistical analysis was performed using SPSS (IBM^®^ SPSS Statistics^®^, Version 29.0.2.0 (20), Armonk, NY, USA). Patients were divided into two groups based on the presence of a mental disorder. Descriptive analyses were performed and presented as mean with standard deviation (SD), or frequencies with percentages when appropriate. For comparison of categorical variables, a Chi-square test was used. For continuous outcomes, a *t*-test was used. We also evaluated and accounted for high levels of collinearity using a correlation matrix. No variables demonstrated a correlation coefficient exceeding 0.7, suggesting that collinearity was not a significant concern. Significant results, from the Chi-squared test performed for categorical variables, were included in a multivariable logistic analysis model. The effect of each risk factor was reported using odds ratios (ORs), 95% confidence intervals (CIs), and *p*-value. By convention, the level of significance was set at *p* < 0.05.

This retrospective study utilizes data from the 2020 Nationwide Readmission Database (NRD), Healthcare Cost and Utilization Project (HCUP), Agency for Healthcare Research and Quality. Therefore, no approval from an institutional or national research committee was needed.

## 3. Results

A total of 6139 patients met our inclusion criteria. A mental health disorder was documented for 3771 patients (61.4%). The 90-day all-cause readmission rate was determined to be 35% (Table 1). 

The subgroup analysis is summarized in Table 2 and reflects the patients’ demographics relative to the presence of MHDs. We found several significant differences between the groups, as follows: patients with MHDs were more likely to be males (*p* = 0.048) and younger in age (56.6 years to 65.7 years, *p* < 0.001) compared to those without mental health disorders. Additionally, MHD patients had significantly longer lengths of stay and higher rates of non-elective admissions.

**Figure 1:**
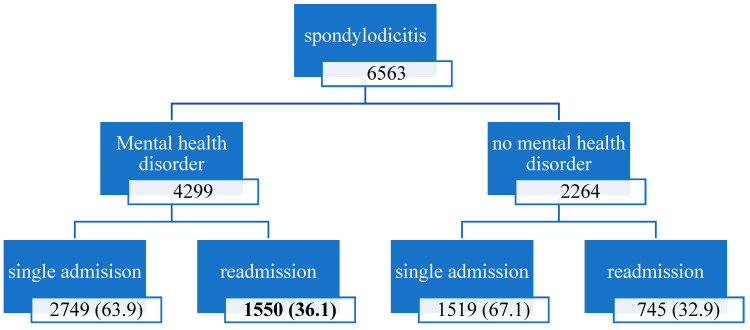
Distributions of readmissions relative to the presence of MHDs. The readmission rate of patients with mental health disorders is significantly higher compared to those without an MHD. Percentages were calculated for each group.

Distributions of readmissions relative to the presence of MHDs and care rendered are visualized in Figure 1. The readmission rate was significantly higher in those with MHDs (36.1% compared to 33.2%), with shorter times until readmission (34.9 days to 37.21 days). The Elixhauser comorbidity indices for both in-hospital mortality and 30-day readmission showed significant differences between the groups (*p* < 0.001), indicating a higher comorbidity burden for MHD patients. The rates for in-patient mortality were similar and did not differ statistically.

To be comprehensive, we included twenty different mental health disorders in our analysis (Table 3). The three most prevalent MHDs were tobacco-related disorders (28.4%), anxiety and fear-related disorders (18.4%), and opioid-related disorders (17.6%).

Substance-related disorders were particularly common, with tobacco (28.4%), opioid (17.6%), and alcohol-related disorders (5.4%) representing a significant proportion of MHD cases. Additionally, anxiety (18.4%), depressive disorders (17.3%), and bipolar disorders (4.6%) were frequently documented. In contrast, schizophrenia, neurocognitive disorders, and personality disorders were less prevalent in this cohort. Other specific MHDs such as schizophrenia and neurocognitive or personality disorders were less common. Overall, bipolar disorders, as well as alcohol, opioid, tobacco and other specified substance-related disorders were significantly more common in patients who were readmitted.

The multivariate regression analysis revealed opioid- and alcohol-related disorders (OR 1.187, *p* = 0.019; OR 1.310, *p* = 0.020) as independent risk factors (Table 4). A slightly increased odds ratio for bipolar, tobacco, and other specified substance-related disorders was observed, but did not reach statistical significance. 

## 4. Discussion

The incidence of two distinct disease entities, spondylodiscitis and mental health disorders, are of rising global concern [1,2,4,24]. In our study we sought to identify the concomitance of these two entities using readmission data from a large national sample. Overall, we identified a 90-day all-cause readmission rate for spondylodiscitis of 35%. A total of 61.4% of those patients diagnosed with SD also suffered from an MHD. Patients with MHDs had a significantly higher readmission rate (36.1%) compared to those without (33.2%). Furthermore, the mean time until readmission in the MHD groups was shorter (34.9 days) as well. Non-elective admission was predominant in both groups, which again underscores the severity of SD.

Beyond spine surgery, a number of studies have consistently shown adverse effects of MHDs on surgical and medical treatment. Patients suffering from mental health disorders are reported to have an increased likelihood of suffering more complications, with increased lengths of stay and having more irregular post-discharge dispositions. Across specialties, MHD patients were found to require more frequent unplanned readmissions and suffer from inferior outcomes [13,15,25,26,27]. The results of our study indeed are in keeping with the existing literature, highlighting potential additional care considerations for treating patients with MHDs, and adding more realistic covariables to quality-of-care expectations in regard to length of stay and readmissions. 

Various previous studies described poorer outcomes, lower functionality and quality of life scores, increased opioid usage, and increased revision rates for patients suffering from depression. High incidences of depression and anxiety disorders have been reported in patients treated for SD. Additionally, a significantly lower quality of life score for surgically treated patients was observed compared to the normal population. These findings suggest that the long and extensive treatments required for SD may further adversely impact MHDs in this vulnerable population [12,13,14,28].

Our analysis shows that five different MHDs were significantly more prevalent in the SD-readmission group (tobacco-, alcohol-, and opioid-related disorders, along with other substance-related disorders and bipolar disorders). Although highly prevalent, depression and anxiety disorders did not differ significantly within our two study cohorts. These findings are in alignment with other published results, showing no effect of these MHDs in postoperative functional outcomes after thoracolumbar surgery. However, Holbert et al. showed that these conditions were associated with more frequent emergency department visits and complications [29]. In addition, anxiety and depression have been shown to influence the quality of life in patients with ankylosing spondylitis and spondydiscitis [19,30]. This suggests that associated behavioral patterns and individual temperament profiles may affect quality of life more than readmission rates.

The psychoneuroimmunological effects of depression have been described. While causal pathways are yet to be understood in full detail, several studies have supported an interactional relationship between depression and rates of infection. Such effects can weaken the immune system, making the patient more susceptible to infections [16,17,31]. A dose–response relationship has been described between depressive symptom severity and mild-infection frequency [18]. Additionally, coping mechanisms such as smoking or abuse of other substances may further influence immune response [31]. However, these psychosocial and psychoneuroimmunological effects are yet to be fully understood.

Although not reaching significance, an increased odds ratio for bipolar disorders as an independent risk factor was observed. However, it was more frequent in readmitted patients (*p* = 0.026). Other disorders, such as schizophrenia or personality disorders, did not differ significantly. These results are partially consistent with other previous studies. Bipolar disorder and schizophrenia are severe mental health disorders with distinct clinical presentations that may influence patient outcomes differently. Bipolar disorder is characterized by episodic mood disturbances, including manic and depressive phases, which may lead to impulsivity, inconsistent healthcare adherence, and an overall increased risk of hospitalization. In contrast, schizophrenia is associated with cognitive impairment, delusions, and disorganized behavior, which can contribute to difficulties in medication compliance, self-care, and access to healthcare resources [32,33,34,35,36]. Menendez et al. showed that schizophrenia, among other MHDs, significantly increased the likelihood of perioperative adverse effects [15]. Additionally, an overall increased mortality for MHDs has been reported [37]. Despite not being independent risk factors for readmission, these specific MHDs still impact patient outcomes and suggest that additional factors, such as treatment adherence, access to psychiatric care, and medical comorbidities, may be crucial in determining patient outcomes. 

Dependency disorders such as alcohol and opioid related disorders were clearly identified as independent risk factors for readmission. The existence of an alcohol-related disorder was the strongest predictor for readmission (OR 1.310; *p* = 0.020). Opioid-related disorders have previously been recognized as a risk factor for SD [4,11]. Although alcohol abuse is not reported as a traditional risk factor for SD, it does impart adverse effects on its host, such as immunosuppression, hypoproteinemia, and increased bleeding tendencies [38]. These substances impair patients through multifactorial pathways and interactions, leading to increased susceptibility to infections and subsequent chronic infection states [39].

This complexity in dependency related disorders may lead to poor compliance and lack of follow-up appointments. Increased odds for readmission were observed for patients with tobacco dependency, though not reaching significancy (OR1.053; *p* = 0.420). Previously, tobacco usage was linked to poorer outcomes and a higher risk of complications in degenerative spine surgery, resulting in increased readmission rates [40]. Although previously identified as a risk factor, tobacco-related disorders may not be as relevant in spondylodiscitis.

It is noted that, in addition to MHDs, multiple factors contribute to the likelihood of hospital readmission in patients with spondylodiscitis. These may also act as confounding variables in this analysis. Medical comorbidities, such as diabetes mellitus, chronic kidney disease, and cardiovascular disorders, have been shown to increase readmission risk and mortality [4,7,10]. Furthermore, treatment algorithms also have a significant impact on the readmission rate, with surgery being protective of readmissions, indicating lower readmission rates and longer times until readmission [41].

There are several limitations to this study impacting findings and interpretation. The use of large databases, such as the National Readmission Database by the Healthcare Cost and Utilization Project (HCUP), may introduce inherent biases due to variations in coding, data selection, and lack of detailed clinical information (such as infection severity, causative pathogens, and treatment modalities). This may lead to underestimation or misclassification of certain psychiatric conditions. Furthermore, substance-related disorders may be more consistently recorded compared to personality, mood, and anxiety disorders due to their direct medical implications, causing misrepresentation of this group. Overall, these factors may limit the availability and accuracy of clinical data, and thus cause misestimation of the readmission rate and associated factors. The readmission rate might be underestimated, as patients can only be tracked within one state. Additionally, data from 2020 may have been influenced by the global COVID-19 pandemic. The pandemic significantly altered clinical practices in a multifactorial way. Among other reasons, this may introduce a potential selection bias. Overall, potential effects will take years to clarify.

## 5. Conclusions

Worldwide incidences of both MHDs and SD are rising. Various negative effects of MHDs on patient outcomes have been described. MHDs are common in patients with SD. Overall, we established a 90-day all-cause readmission rate of 35%. Patients suffering from an MHD were significantly more likely to experience a readmission than those without an MHD. Furthermore, the mean time until readmission was shorter in this group. Our analysis identified alcohol- and opioid-related disorders as independent risk factors for readmission. Although bipolar disorders were more frequent among readmitted patients, this did not reach significance as a risk factor. Other, common disorders like depression and anxiety did not affect the rate of readmission. Identification of high-risk patients may allow for targeted interventions, such as psychiatric consultation, addiction treatment referrals, and structured post-discharge follow-ups to reduce readmission risk. Future research into direct and indirect effects of MHDs in the context of SD management outcomes as well as the development of risk stratification models may be beneficial.

## Figures and Tables

**Table 1:** General study population.

	Spondylodiscitis n (%)
General study population	6139
Mental health disorder	3771 (61.4)
Readmission rate	2146 (35)

**Table 2:** Demographics of the study population. Bold values indicate statistical significance.

	Mental Health Disorder n = 3771	No Mental Health Disorder n = 2368	*p*-Value
Patient Demographics	n (%) or	Mean (±SD)	
Age	56.56 (±15.78)	65.70 (±14.19)	**<0.001**
Male	2234 (59.2)	1463 (61.8)	**0.048**
Length of stay	12.67 (±14.85)	10.19 (±13.14)	**<0.001**
Non-elective admission	3575 (94.8)	2187 (92.4)	**<0.001**
Readmission	1360 (36.1)	786 (33.2)	**0.022**
Time to first readmission (days)	34.90 (±22.94)	37.21 (±23.01)	**0.025**
Elixhauser in-hospital mortality index	−1.87 (±9.2)	1.56 (±8.94)	**<0.001**
Elixhauser 30-day readmission index	5.81 (±5.29)	5 (±6.11)	**<0.001**
In-hospital death	44 (1.17)	29 (1.2)	0.843

**Table 3:** Mental health disorders. Bold values identify statistical significance.

Mental Health Disorders	n = 6139 (%)	*p*-Value
Neurocognitive disorders	414 (6.74)	0.939
Schizophrenia spectrum and other psychotic disorders	128 (2.09)	0.325
Other specified and unspecified mood disorders	61 (0.99)	0.930
Depressive disorders	1062 (17.3)	0.124
Bipolar and related disorders	282 (4.59)	**0.026**
Anxiety and fear-related disorders	1131 (18.42)	0.855
Miscellaneous mental and behavioral disorders/conditions	11 (0.18)	0.922
Personality disorders	40 (0.65)	0.995
Alcohol-related disorders	332 (5.41)	**0.005**
Mental and substance use disorders in remission	191 (3.11)	0.731
Opioid-related disorders	1079 (17.58)	**0.001**
Cannabis-related disorders	269 (4.38)	0.237
Sedative-related disorders	54 (0.88)	0.543
Stimulant-related disorders	515 (8.39)	0.441
Tobacco-related disorders	1746 (28.44)	**0.013**
Other specified substance-related disorders	319 (5.2)	**0.047**
Obsessive-compulsive and related disorders	18 (0.29)	0.885
Trauma- and stressor-related disorders	226 (3.68)	0.477
Disruptive, impulse-control and conduct disorders	13 (0.212)	0.369
Neurodevelopmental disorders	108 (1.76)	0.800

**Table 4:** Multivariate regression analysis. Bold values identify statistical significance.

	Odds Ratio	95% Confidence Interval	*p*-Value
Bipolar and related disorders	1.219	0.952–1.561	0.117
Alcohol	1.310	1.044–1.644	**0.020**
Opioid-related disorders	1.187	1.029–1.371	**0.019**
Tobacco-related disorders	1.053	0.929-1.195	0.420
Other specified substance-related disorders	1.163	0.917-1.475	0.213

## Data Availability

The dataset is publicly available from the Healthcare Cost and Utilization Project (HCUP), Agency for Healthcare Research and Quality.

## Abbreviations

The following abbreviations are used in this manuscript:

SD	Spondylodiscitis
MHD	Mental health disorder
NRD	Nationwide Readmission Database
CCSR	Clinical classifications software refined
LOS	Length of stay
HCUP	Healthcare Cost and Utilization Project
ICD-10	International Classification of Diseases, Tenth Revision, Clinical Modification
